# Notch signaling in mouse blastocyst development and hatching

**DOI:** 10.1186/s12861-020-00216-2

**Published:** 2020-06-02

**Authors:** Mariana R. Batista, Patrícia Diniz, Ana Torres, Daniel Murta, Luís Lopes-da-Costa, Elisabete Silva

**Affiliations:** 1 Reproduction and Development Laboratory, CIISA – Centro de Investigação Interdisciplinar em Sanidade Animal, Faculdade de Medicina Veterinária, Universidade de Lisboa, 1300-477 Lisbon, Portugal; 2 CBIOS – Research Centre for Biosciences and Health Technologies, Faculty of Veterinary Medicine, Lusófona University of Humanities and Technologies, Lisbon, Portugal

**Keywords:** Blastocyst, Development, Hatching, Notch, Mouse

## Abstract

**Background:**

Mammalian early embryo development requires a well-orchestrated interplay of cell signaling pathways. Notch is a major regulatory pathway involved in cell-fate determination in embryonic and adult scenarios. However, the role of Notch in embryonic pre-implantation development is controversial. In particular, Notch role on blastocyst development and hatching remains elusive, and a complete picture of the transcription and expression patterns of Notch components during this time-period is not available.

**Results:**

This study provided a comprehensive view on the dynamics of individual embryo gene transcription and protein expression patterns of Notch components (receptors Notch1–4; ligands Dll1 and Dll4, Jagged1–2; and effectors Hes1–2), and their relationship with transcription of gene markers of pluripotency and differentiation (*Sox2*, *Oct4*, *Klf4*, *Cdx2*) during mouse blastocyst development and hatching. Transcription of *Notch1–2*, *Jagged1–2* and *Hes1* was highly prevalent and dynamic along stages of development, whereas transcription of *Notch3–4*, *Dll4* and *Hes2* had a low prevalence among embryos. Transcription levels of *Notch1*, *Notch2*, *Jagged2* and *Hes1* correlated with each other and with those of pluripotency and differentiation genes. Gene transcription was associated to protein expression, except for Jagged2, where high transcription levels in all embryos were not translated into protein. Presence of Notch signaling activity was confirmed through nuclear NICD and Hes1 detection, and downregulation of *Hes1* transcription following canonical signaling blockade with DAPT. In vitro embryo culture supplementation with Jagged1 had no effect on embryo developmental kinetics. In contrast, supplementation with Jagged2 abolished *Jagged1* transcription, downregulated *Cdx2* transcription and inhibited blastocyst hatching. Notch signaling blockade by DAPT downregulated transcription of *Sox2*, and retarded embryo hatching.

**Conclusion:**

Transcription of Notch genes showed a dynamic pattern along blastocyst development and hatching. Data confirmed Notch signaling activity, and lead to the suggestion that Notch canonical signaling may be operating through Notch1, Notch3, Jagged1 and Hes1. Embryo culture supplementation with Jagged1 and Jagged2 unveiled a possible regulatory effect between Jagged1, Cdx2 and blastocyst hatching. Overall, results indicate that a deregulation in Notch signaling, either by its over or under-activation, affects blastocyst development and hatching.

## Background

Abnormal mammalian preimplantation embryo development is responsible for a significant prevalence of embryo-fetal mortality in both human and domestic animal species [1, 2]. However, the complex spatial and temporal orchestration of cellular events associated with early development, which require a finely tuned inter-cellular communication, is still largely unresolved. Zygote cleavage leads to the compact morula stage, where the first cellular differentiation events originate the blastocyst [3]. The blastocyst comprises two cell types: i) trophectoderm (TE) – which will give rise to the placenta, and ii) inner-cell-mass (ICM) – which will constitute the embryo itself [4]. The maintenance of TE epithelial integrity and differentiated status relies on transcription factor Cdx2 expression [5, 6]. Likewise, ICM pluripotency maintenance relies on expression of a wide network of transcription factors, namely Sox2, Oct4 and Klf4 [7, 8].

Several cell signaling pathways critical for embryo development have been identified in the mouse preimplantation embryo [9]. The Notch cell signaling pathway, highly conserved among invertebrates and vertebrates, has been implicated as a main regulator of cellular differentiation and proliferation in many adult and embryonic scenarios [10–13], and was identified in several mammalian preimplantation embryos, including the mouse [14–19]. In mammals, Notch is a receptor-ligand based cell signaling pathway composed of four receptors (Notch1–4) and five ligands (Delta-like (Dll) 1, 3 and 4; Jagged1 and 2). Notch signaling may be conveyed in the so-called canonical and non-canonical forms, reflecting a high mechanistic complexity, yet to be fully understood (for reviews see [20–22]). Briefly, the canonical signaling results from the interaction of a ligand expressed by the signal-sending cell with a transmembrane receptor expressed by a signal-receiving neighboring cell. This binding of the ligand in *trans* leads to the sequential cleavage of the intracellular domain (NICD) of the receptor by extracellular ADAM proteases and an intracellular γ-secretase, and its translocation to the nucleus. Here, NICD de-represses the transcription complex Rbpj, to regulate the transcription of Notch effector genes (including *Hes1* and *Hes2*). Signal termination is ensured by ubiquitin-dependent proteasome degradation of NICD. Knowledge on non-canonical Notch signaling in mammalian systems is still largely fragmentary [22]. This form is ligand independent and/or does not require NICD interaction with Rbpj [23]. The role of Notch signaling in embryo preimplantation development is controversial. Earlier studies reported that canonical Notch signaling is not required for early embryo development [24, 25], although subsequent studies showed that pharmacological inhibition of the pathway with DAPT (a γ-secretase inhibitor) affects embryo implantation [26]. More recently, studies using mutant knockout embryos unveiled a role for Notch, together with the Hippo pathway, on TE lineage assignment [13, 27, 28].

This study considered the evaluation of Notch signaling, in individual embryos, in a defined time-frame of mouse preimplantation embryonic development – blastocyst differentiation from compact morulae until blastocyst hatching. This evaluation included gene transcription (quantitative real-time PCR; qRT-PCR), protein expression (immunocytochemistry; ICC), and in vitro embryo culture supplementation with Notch activators and inhibitors. In transcription analysis, the first step was to identify the prevalence of transcription of Notch (receptors, ligands and effectors) and pluripotency and differentiation genes along four developmental stages: compact morulae (CM), blastocyst (BL), expanded blastocyst (EBL) and hatched blastocyst (HBL). The second step was to evaluate the levels of transcription of each gene at each developmental stage. The above data allowed the evaluation of transcription relationships (correlations) between Notch and pluripotency and differentiation genes. Evaluation of protein expression by ICC at the BL stage evidenced mRNA translation and the nuclear identification of NICD and/or effectors, thus confirming Notch signaling activity. Finally, in vitro embryo culture with a γ-secretase (Notch signaling blockade) or with Notch ligands (putative activators) evidenced phenotypic effects in embryo development and gene transcription. Therefore, the objectives of this study were, at the individual embryo level, to evaluate i) the signaling status of Notch pathway and the dynamic patterns of transcription and expression of Notch components, from the compact morulae stage until blastocyst hatching; ii) the relationship between the transcription of Notch components and gene markers of embryonic pluripotency and differentiation; and iii) the effects of supplementation with Notch ligands and Notch signaling inhibitors on blastocyst development and hatching.

## Results

### Gene transcription

**Fig. 1:**
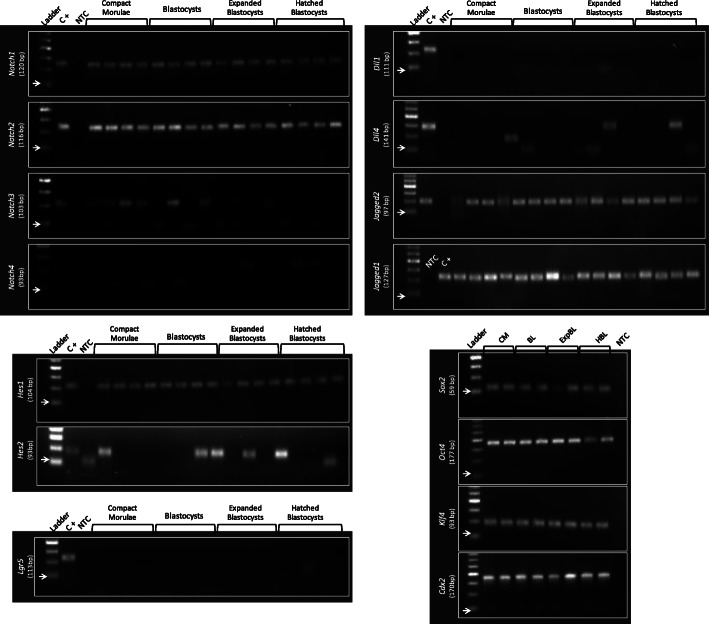
Agarose gels of qRT-PCR products. For each Notch component gene (receptors, ligands and effectors; plus negative control), four representative embryos of each developmental stage (3.5 dpc compact morulae, blastocysts and expanded blastocysts, and 4.5 dpc hatched blastocysts) are shown. For each pluripotency and differentiation gene markers, two representative embryos of each developmental stage are shown. Ladder: DNA ladder with 50 bp increments; the arrow (→) signals the 50 bp mark; C+: positive control gene; for each analyzed gene, a tissue sample known to transcribe the analyzed gene was added, and the qRT-PCR reaction product added in the gel (see Methods section for details); NTC: non-template control

Transcription prevalence and levels of Notch and pluripotency and differentiation genes was analyzed by qRT-PCR in individual embryos at four developmental stages: 3.5 days *post-coitum* (dpc) CM, BL and EBL, and 4.5 dpc HBL. Based on RNA-seq databases [18, 19], *Lgr5* was chosen as negative gene transcription control, as this pluripotency-associated gene showed very low transcription levels in embryonic cells of developmental stages considered in this study. Table 1 shows gene transcription prevalence among individual embryos and stages of development, and Fig. 1 illustrates the respective agarose gels of qRT-PCR products (displaying four embryos / gene / stage of development). Regarding Notch genes, transcription of receptors *Notch1* and *Notch2*, ligand *Jagged2* and effector *Hes1* was detected in all embryos, and transcription of ligand *Jagged1* was detected in all but four embryos. Receptors *Notch3* and *Notch4*, ligand *Dll4* and effector *Hes2* had inconsistent transcription among embryos, whereas transcription of ligand *Dll1* was not detected. Transcription of pluripotency and differentiation genes (*Sox2*, *Oct4*, *Klf4*, *Cdx2*) was detected in all embryos, whereas transcription of negative control *Lgr5* was not detected.

**Table 1:** Prevalence of gene transcription among embryos at each stage of development

Gene	Stage of development			
	CM	BL	EBL	HBL
*Notch1*	9/9	9/9	7/7	5/5
*Notch2*	9/9	9/9	7/7	5/5
*Notch3*	5/9	3/9	0/7	2/5
*Notch4*	0/9	0/9	1/7	2/5
*Dll1*	0/9	0/9	0/7	0/5
*Dll4*	0/9	0/9	2/7	1/5
*Jagged1*	6/9	9/9	7/7	4/5
*Jagged2*	9/9	9/9	7/7	5/5
*Hes1*	9/9	9/9	7/7	5/5
*Hes2*	5/9	3/9	3/7	0/5
*Lgr5*	0/9	0/9	0/7	0/5
*Sox2*	9/9	9/9	7/7	5/5
*Klf4*	9/9	9/9	7/7	5/5
*Cdx2*	9/9	9/9	7/7	5/5
*Oct4*	9/9	9/9	7/7	5/5

Prevalence is depicted as the number of embryos with specific amplification of the gene in relation to the total number of embryos analyzed. *CM* Compact Morulae, *BL* Blastocyst, *EBL* Expanded Blastocyst, *HBL* Hatched Blastocyst

**Fig. 2:**
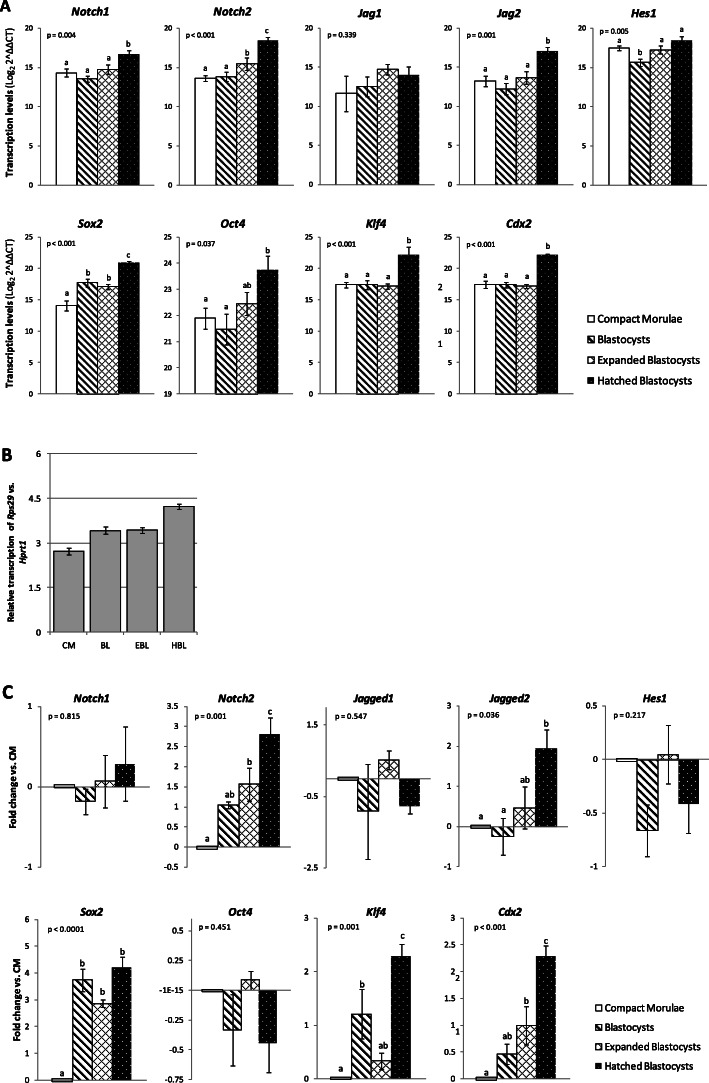
Transcription of Notch components and pluripotency and differentiation gene markers in mouse early embryonic development. Quantitative real-time (qRT-PCR) was used to detect and quantify the presence of transcripts in 3.5 dpc compact morulae (*n* = 9), blastocysts (*n* = 9) and expanded blastocysts (*n* = 7), and in 4.5 dpc hatched blastocysts (*n* = 5). Analyzed genes (most prevalent): Notch receptors – *Notch1* and *Notch2*; Notch ligands – *Jagged1* and *Jagged2*; Notch effectors – *Hes1*; Pluripotency and differentiation marker genes - *Sox2*, *Oct4*, *Klf4* and *Cdx2*. Bars represent mean transcription levels ± s.e.m. ANOVA *p* values are indicated for each gene analysis. Bars with different letters differ significantly (post-hoc LSD). **a:** For data analysis, Ct values were normalized to housekeeping gene 1 (*Rps29*) and the ∆Ct values obtained further calibrated with housekeeping gene 2 (*Hprt1*), generating ∆∆Ct values. These values were log transformed and results presented as the Log_2_ of power of ∆∆Ct values. **b:** Log_2_ of power of ∆Ct values of transcription levels of housekeeping genes *Rps29* and *Hprt1* at each developmental stage; CM = Compact Morulae; BL = Blastocyst; EBL = Expanded Blastocyst; HBL = Hatched Blastocyst. **c**: For data analysis, Ct values of each target gene were normalized with the mean Ct values of housekeeping genes *Rps29* and *Hprt1*, and the obtained ∆Ct values were then calibrated to ∆Ct values of compact morulae (shown as 0.0), originating the ∆∆Ct values for log transformation. Results are also presented as the Log_2_ of power of ∆∆Ct values

**Fig. 3:**
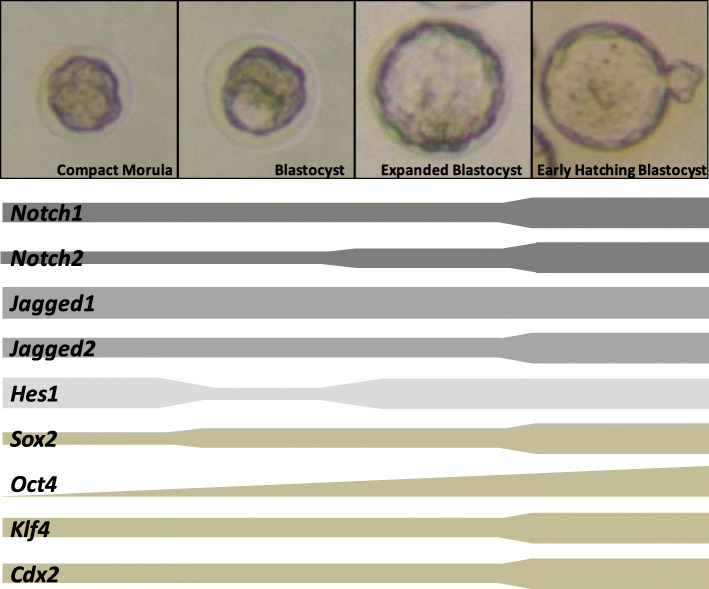
Schematic illustration of the dynamic transcription patterns of Notch and pluripotency and differentiation genes along mouse early embryonic development

Figure 2 panel a, shows the mean transcription levels of Notch and pluripotency and differentiation genes at each developmental stage (values are presented as the Log_2_ of power of ∆∆Ct values). Only genes with consistent transcription among embryos were considered in this analysis. Figure 2 panel b shows the fold change values of transcription levels of *Rps29* and *Hprt1* control endogenous (housekeeping) genes at each developmental stage. The transcription levels of target genes at the BL, EBL and HBL stages were then compared to those at the CM stage (values are presented as the Log_2_ of power of ∆∆Ct values, with CM stage as calibrator) (Fig. 2 panel c). Based on above results, the dynamics of gene transcription along developmental stages is schematically illustrated in Fig. 3. As depicted from these figures, transcription of *Sox2*, *Oct4*, *Klf4*, *Cdx2*, *Notch1*, *Notch2* and *Jagged2* increased throughout development, mainly at the HBL stage, whereas transcription of *Jagged1* and *Hes1* remained fairly constant.

Transcription levels of *Notch1*, *Notch2*, *Jagged2* and *Hes1* correlated with those of all pluripotency and differentiation genes (*r* = 0.72 to 0.95, *p* = 0.004 to *p* < 0.0001). *Notch1* correlated with *Notch2*, *Jagged2* and *Hes1* (*r* = 0.75 to 0.86, *p* < 0.0001), *Notch2* correlated with *Jagged2* and *Hes1* (*r* = 0.79 and 0.72, *p* < 0.0001 and *p* = 0.001, respectively), and *Jagged2* correlated with *Hes1* (*r* = 0.78, *p* < 0.0001).

### Gene expression

**Fig. 4:**
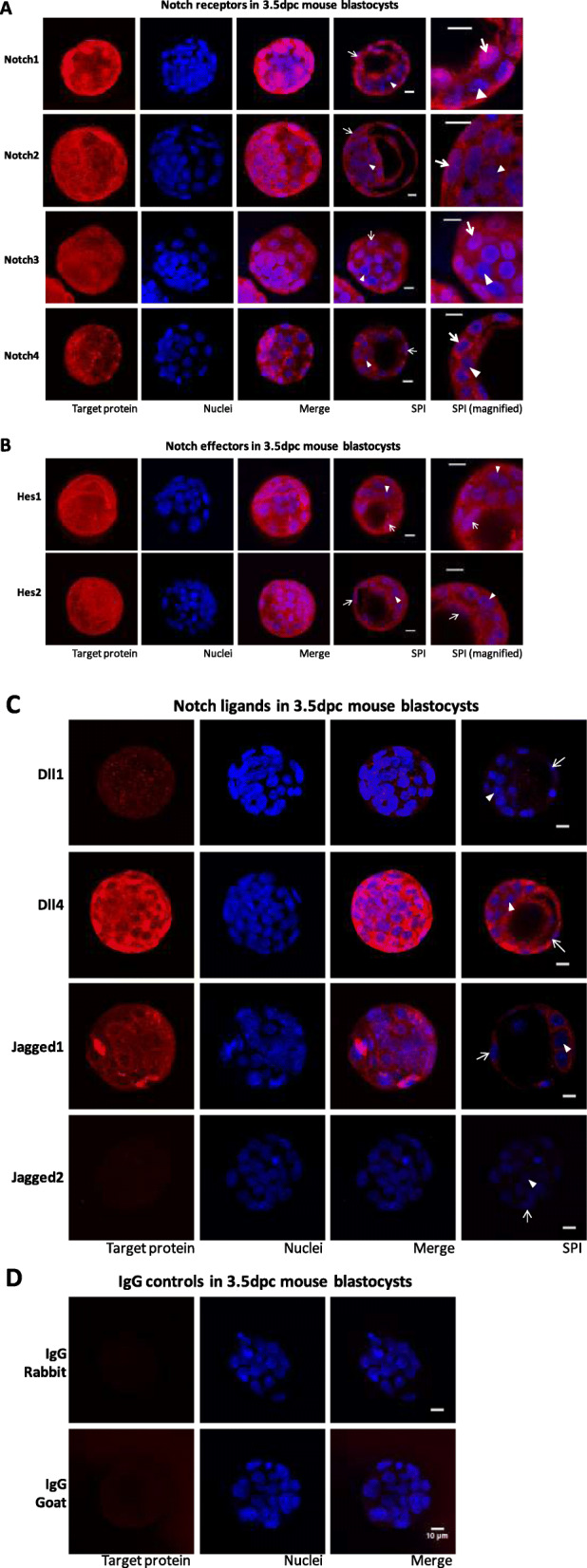
Expression of Notch receptors Notch1–4 (**a**), Notch effectors Hes1–2 (**b**), Notch ligands Delta-like1 and 4 and Jagged1–2 (**c**), and negative controls (Rabbit and Goat IgG; **d**) in 3.5 dpc blastocysts. Confocal photomicrographs show representative images of each target protein immunostaining. Images were selected to show the similar staining pattern of six blastocysts, for each protein. Target proteins are stained red and nuclei are stained blue with Hoechst. Images in the first three columns are maximum intensity projections of the obtained Z-stack; the fourth and fifth columns are representative single plane images (SPI). Examples of presumptive trophectoderm cells’ nuclei are marked with arrows (→) and examples of presumptive inner cell mass cells’ nuclei are marked with arrowheads (►). Scale bar 10 μm. Notice that there is no detectable staining for Dll1 and Jagged2 proteins, which show a similar staining to that of negative controls

Since the BL represents the earliest developmental stage in which the two initial cell lineages – ICM and TE – are segregated and have reached their final spatial location, this embryonic stage was chosen to evaluate the presence of Notch proteins. As shown in Fig. 4a, Notch1–4 were expressed in BL, and Notch1 and Notch3 were detected in the nucleus of presumptive TE cells. This indicates that the receptors were cleaved and NICD was translocated into the nucleus, thus confirming Notch signaling activation through these receptors. Ligands Dll4 and Jagged1 were expressed in BL, whereas ligands Dll1 and Jagged2 were not detected (Fig. 4c). Effector Hes1 was detected in the nucleus of some cells, whereas Hes2 only showed a diffuse pattern in the cytoplasm (Fig. 4b).

### Notch signaling activation or blockade in cultured embryos

**Fig. 5:**
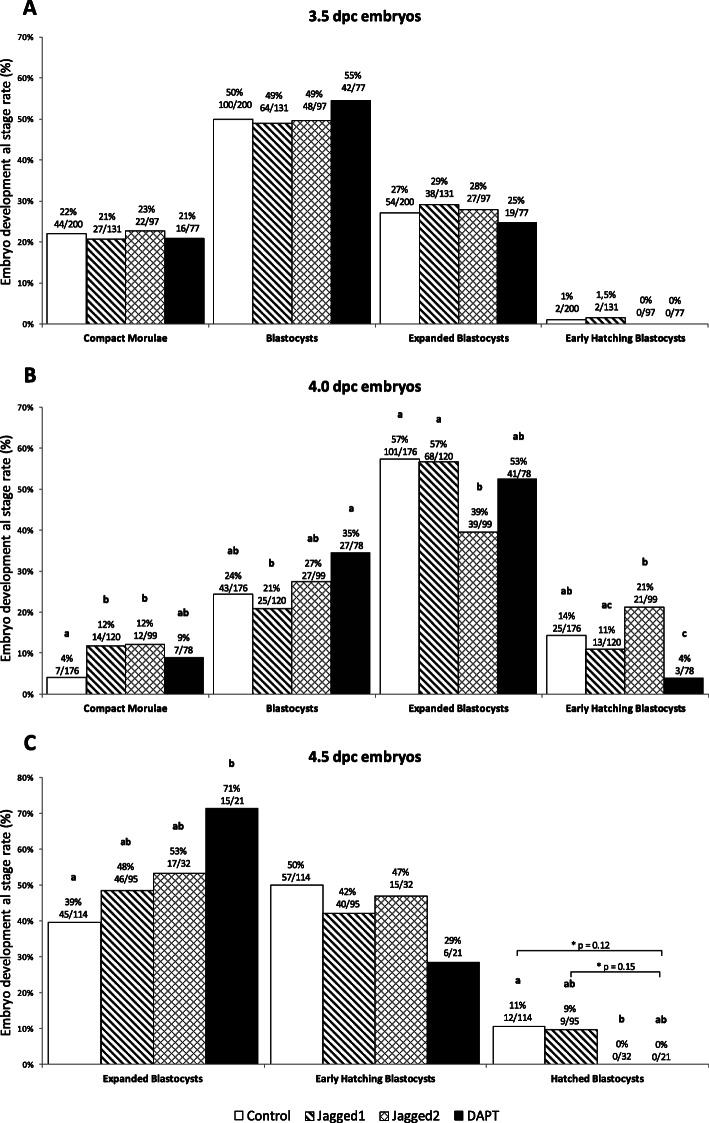
Effect of pharmacological Notch signaling inhibition and activation on mouse embryo developmental kinetics. Mouse 2.5 dpc embryos were in vitro cultured in the presence of a Notch inhibitor (DAPT) or Notch ligands Jagged1 and Jagged2 until 4.5 dpc. Embryos were observed after 24 h in culture (at 3.5 dpc; **a**), 36 h in culture (at 4.0 dpc; **b**) and 48 h in culture (at 4.5 dpc; **c**) and morphologically evaluated. In vitro culture of a subset of embryos was discontinued at 3.5 dpc or 4.0 dpc to perform transcription analysis. Numbers above bars indicate the number of viable embryos in culture / the number of total embryos. Different letters within the same developmental stage differ significantly, *p* < 0.05 (Chi-square test). Asterisks (*) indicate the exact *p* value of the Chi-square test

To further confirm Notch activity in mouse early embryonic development, Notch signaling was inhibited through a pharmacological approach with DAPT (N-[N-(3,5-Difluorophenacetyl)-L-alanyl]-S-phenylglycine t-butyl ester), a γ-secretase inhibitor which prevents the intracellular cleavage of NICD and its translocation to the nucleus. Together with embryo culture supplementation with recombinant Jagged1 and Jagged2 (putative Notch activators), this experiment allowed the observation of effects of Notch signaling inhibition or activation on blastocyst development and hatching. As shown in Table 2, DAPT, Jagged1 and Jagged2 treatments had no effect on embryo viability, as depicted from the number of non-degenerated morphologically normal embryos progressing in culture. However, embryo kinetics was affected by DAPT treatment, which decreased the early hatching blastocyst rate at 4.0 dpc (*p* < 0.05), and the hatched blastocyst rate at 4.5 dpc (statistical tendency, *p* = 0.12) (Fig. 5b-c). At 4.0 dpc, both Jagged1 and Jagged2 treatments prevented the progression of CM (Fig. 5b), whereas at 4.5 dpc Jagged2 supplementation significantly inhibited blastocyst hatching (Fig. 5c).

**Table 2:** Effect of embryo culture supplementation with DAPT, Jagged1 and Jagged2 on mouse embryo survival

Group	n	3.5 dpc embryos n (%)	n	4.0 dpc embryos n (%)	n	4.5 dpc embryos n (%)
**Control**	216	200 (93%)	192	176 (92%)	127	114 (90%)
**DAPT**	86	77 (90%)	88	78 (89%)	26	21 (81%)
**Jagged1**	146	131 (90%)	128	120 (94%)	108	95 (88%)
**Jagged2**	102	97 (95%)	103	99 (96%)	40	32 (80%)

Columns marked as **n** show the total number of embryos present in culture; columns marked as 3.5 dpc, 4.0 dpc and 4.5 dpc show the number of non-degenerated morphologically normal embryos progressing in culture up to that time-point

**Fig. 6:**
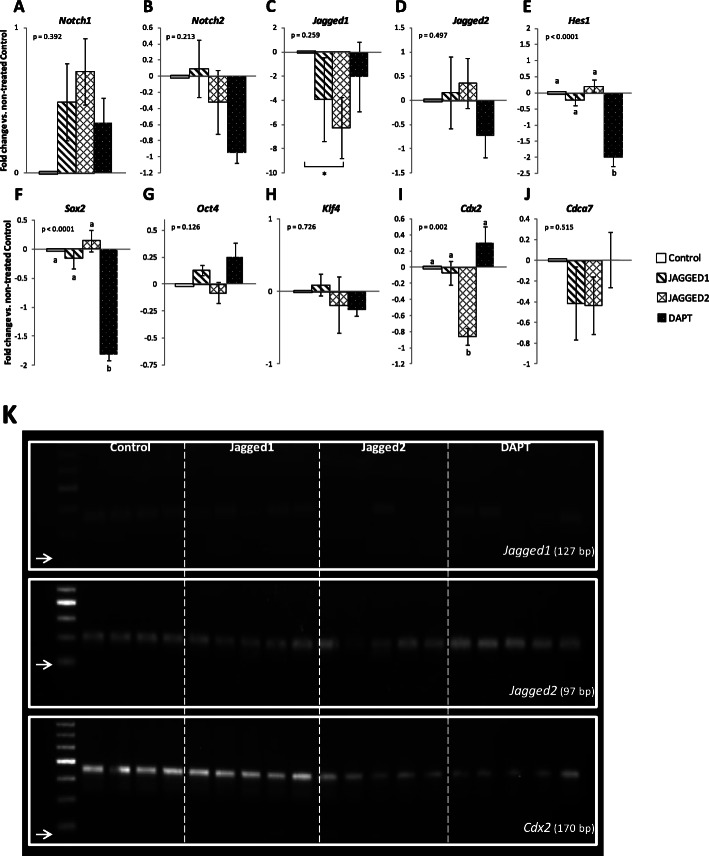
Effect of pharmacological Notch signaling inhibition and activation on gene transcription in 4.0 dpc mouse expanded blastocysts. Mouse 2.5 dpc embryos were in vitro cultured in the presence of a Notch inhibitor (DAPT) or of Notch ligands Jagged1 and Jagged2, for 36 h, until 4.0 dpc. Expanded blastocysts from groups Control (*n* = 5), Jagged1-treated (*n* = 5), Jagged2-treated (*n* = 5) and DAPT-treated (*n* = 6) were processed for qRT-PCR analysis. **a-j:** Transcription of *Notch1* (**a**), *Notch2* (**b**), *Jagged1* (**c**), *Jagged2* (**d**) and *Hes1* (**e**), and of pluripotency and differentiation genes *Sox2* (**f**), *Oct4* (**g**), *Klf4* (**h**), *Cdx2* (**i**) and *Cdca7* (**j**) were analyzed. Bars represent Log_2_ of power of ∆∆Ct values. These values were generated by first normalizing the Ct values of each target gene with the mean Ct values of the endogenous control genes *Rps29* and *Hprt1*. The obtained ∆Ct values were then calibrated to ∆Ct values of Control embryos, which were used as calibrators (shown as 0.0), originating the ∆∆Ct values for log transformation; error bars show the standard error of the mean (s.e.m). Exact ANOVA results (p) are shown for each gene. Different letters within the same gene represent significantly different mean values (*p* < 0.05; LSD post-hoc). *Transcription of *Jagged1* differs significantly (*p* = 0.038) between groups Control and Jagged2-treated (T-test). **k:** Agarose gels of qRT-PCR products of genes *Jagged1*, *Jagged2* and *Cdx2*. Images illustrate results from representative 4.0 dpc expanded blastocysts from groups Control (*n* = 4), Jagged1-treated (*n* = 5), Jagged2-treated (*n* = 5) and DAPT-treated (*n* = 5). The DNA ladder has 50 bp increments, and the arrow (→) signals the 50 bp mark

To evaluate the possible relationship between the above changes in developmental kinetics and gene transcription, individual EBL of control and treated groups were analyzed by qRT-PCR for transcription of *Notch1, Notch2*, *Jagged1*, *Jagged2*, *Hes1*, *Sox2*, *Oct4*, *Klf4*, *Cdx2*, *Lgr5* and *Cdca7*. Transcription of this latter pluripotency gene (*Cdca7*), regulated by Notch in later embryonic events, such as hematopoietic stem cell emergence [29], was here detected at this earlier stage of development. As shown in Fig. 6 a-j, Notch signaling blockade by DAPT downregulated transcription of *Hes1* and *Sox2* (*p* < 0.0001) and tended to decrease (*p* = 0.06) transcription of *Notch2*. Supplementation with Jagged1 decreased *Jagged1* transcription (although non-significantly) and had no effect on *Jagged2* transcription. In contrast and interestingly, supplementation with Jagged2 although not affecting its own transcription, abolished *Jagged1* transcription in all but one embryo, and downregulated *Cdx2* transcription. The presence of transcripts of *Jagged1*, *Jagged2* and *Cdx2* following treatments with DAPT, Jagged1 and Jagged2 was further confirmed by qRT-PCR product visualization in agarose gels (Fig. 6 k). Additionally, the transcription of the negative control *Lgr5* was not detected.

Correlation analysis showed that control 4.0 dpc EBL showed a positive strong correlation between *Hes1* and *Cdca7* (*r* = 0.98; *p* = 0.005). A similar correlation was found in DAPT treated embryos (*r* = 0.98; *p* = 0.001), but was not present in Jagged1 (*p* = 0.32) and Jagged2 (*p* = 0.32) supplemented embryos.

## Discussion

To the author’s best knowledge, this is the first report on the dynamics of transcription of Notch and of markers of embryonic pluripotency and differentiation genes in individual embryos, from the time of the first cellular differentiation to blastocyst hatching. The results indicate that transcription of Notch components is highly dynamic during mouse blastocyst development and hatching. This approach allowed the assessment of gene transcription relationships, at the individual embryo level, providing so far unique data, not available from studies with pools of embryos or isolated blastomeres. This individual embryo approach revealed that transcription of *Notch1*, *Notch2*, *Jagged1*, *Jagged2* and *Hes1* was ubiquitous from the CM to HBL stages, whereas transcription of *Notch3*, *Notch4*, *Dll4* and *Hes2* was inconsistent along those developmental stages. These transcription patterns of Notch genes partially deviate from those reported by Cormier et al. [14], who evaluated transcription in pools of mouse embryos by nested RT-PCR (inconsistencies between studies in *Notch3*, *Notch4*, *Dll1* and *Dll4* transcription patterns). In the present study, the high accuracy and sensitivity of qRT-PCR, as well as the confirmation of amplicon sequence, allowed for the exclusion of false positives resulting from unspecific amplifications of similar strands of nucleotides, as well as the detection of very small amounts of mRNA copies from single embryos. Recent studies used RNA-Seq analysis of single mouse blastomeres to identify several species of mRNA [18, 19]. Although this is a very useful approach to evaluate overall transcription status of a given cell, a full scan of the whole embryonic cells, especially of more advanced stages, such as HBL which can comprise up to 70 cells [30], is still not available. Since intercellular communication requires the analysis of both the signal sending and signal receiving cells, the loss of information from either of these cells, will provide an incomplete picture of embryonic gene transcription. In fact, in the above studies [18, 19] a low number of copies of *Notch2*, *Dll4, Jagged1, Jagged2* and *Hes2* transcripts were detected, or were not detected at all. This could be due not only to individual embryo variability, as also observed by others [28], but also to the individual blastomere signaling status, which could be in either a signal sending or signal receiving state, since they are mutually exclusive [31].

The presence of transcripts in embryos needs to be interpreted with caution, since an oocyte mRNA pool may be present and be responsible for protein production before the activation of the embryonic genome [32]. Although most of this mRNA pool is translated into protein and degraded during maternal to embryonic transition, which in the mouse occurs mainly at the 2-cell stage [33], up to 10% of maternal mRNA persists until the BL stage [34]. Additionally, cells have post-transcriptional regulating mechanisms that allow them to stock mRNA without immediately translating it into protein [35]. This means that the presence of transcripts may not reflect the protein composition of an embryo at a given stage. In fact, in BL, although *Notch4* and *Dll4* transcripts were not detected, Notch4 and Dll4 proteins were detected. These proteins may have been translated at previous embryonic stages and have not yet been degraded. Inversely, *Jagged2* transcripts were detected in all embryos, but Jagged2 protein was not expressed in BL. At this stage, the embryo may be merely storing *Jagged2* mRNA, which will be translated at a later stage. In fact, the translation of the accumulated *Jagged2* transcripts may only occur at hatching when the embryo enters in direct contact with the endometrium. In this scenario, Jagged2 may be involved, both in the hatching process and in embryo-maternal communication, since several Notch receptors and effectors were identified in the mouse uterine epithelium [36].

*Notch1* transcription was constant until the EBL stage, increasing at the HBL stage, and signaling was activated through this receptor, as the protein was detected in the nucleus. This may indicate that besides a constitutive function [26–28] Notch1 may be regulating other cell functions. Notch3 was also detected in the nucleus of embryonic cells, which indicates that Notch signaling is also being activated through this receptor. On the other hand, as Notch2 remains in the cytoplasm and, since Notch receptors are not redundant [37], results indicate that, at this embryonic stage, only Notch1 and Notch3 are being required. Effector Hes1 was detected in the nucleus, whereas Hes2 only showed a diffuse staining pattern in the cytoplasm. This indicates that Notch signaling may be conveyed through Hes1. In this scenario, as Jagged2 is not expressed at the BL stage, Jagged1 appears as the ligand involved in canonical Notch receptor activation. The diffuse pattern accumulation of Hes2 in the cytoplasm after translation, without translocation to the nucleus, was already observed in other scenarios [38]. This may indicate an additional regulatory mechanism for conveying Notch activity in embryos. Further studies are required to investigate the participation of other Notch effectors, such as the Hey gene family [39], or if Notch signaling is established non-canonically, namely by interacting with other signaling pathways such as Wnt [23] and Hippo [13, 27, 28].

Transcription of embryonic pluripotency and differentiation gene markers followed the patterns previously described by others [15, 18, 40]. The transcription levels of these genes were correlated with those of Notch genes, suggesting that they may be the target of Notch signaling or, conversely, operate to modulate Notch signaling. Menchero et al. [28] showed that Notch is a major activator of *Cdx2* transcription from the 2-cell to the morula stage, but from this stage until the blastocyst stage, *Cdx2* transcription is activated by Hippo. In this study, transcription of *Cdx2* correlated with those of *Notch1*, *Notch2*, *Jagged2* and *Hes1*. Therefore, Notch may still be regulating *Cdx2* transcription at the BL stage.

The presence of Notch signaling activity was further confirmed by the observed downregulation of its effector *Hes1*, following DAPT treatment. This pharmacological blockade of Notch signaling affected embryo developmental kinetics, retarding blastocyst hatching, and downregulated *Sox2* transcription. The above effect on blastocyst hatching was also observed following Jagged2 supplementation. This indicates that a deregulation in Notch signaling, either by its over or under-activation, affects blastocyst development and hatching.

Modulation of Notch signaling through its serrated type ligands has been widely used in many pathological scenarios. The use of anti-Jagged or Jagged overexpression therapies has been extensively studied with varying results [41–43]. Jagged1 supplementation had no major effect on embryo developmental kinetics. This could be due to a sufficient expression of this ligand by the embryo itself, turning supplementation redundant. Interestingly, Jagged2 supplementation abolished *Jagged1* transcription, and this was associated with a downregulation of *Cdx2* transcription and with an impaired blastocyst hatching. This indicates that Jagged2 supplemented embryos had no internal or external source of Jagged1 to maintain a satisfactory *Cdx2* transcription level. This points to a regulatory mechanism by which Jagged1 controls *Cdx2* transcription, and the completion of blastocyst hatching. Alternatively, as Cdx2 is not believed to be an active participant in this process [27], it is possible that *Jagged1* is linked to blastocyst hatching through its interplay with other cell signaling pathways.

Notch signaling activates *Cdca7* transcription in hematopoietic stem cell specification during zebrafish embryonic development [29]. Here, in the mouse model, transcription of *Cdca7* was ubiquitously detected at a much earlier embryonic developmental stage. Transcription of *Cdca7* correlated with that of *Hes1*, in control and DAPT-treated embryos. Both *Hes1* and *Cdca7* have promoters with Rbpj-binding sites, being potential Notch transcriptional targets. However, transcription of *Hes1* was downregulated by DAPT treatment, whereas *Cdca7* transcription was not affected. This may indicate that *Notch* is not regulating *Cdca7* transcription in this mammalian embryonic stage scenario. Nevertheless, the observed significant correlation between *Hes1* and *Cdca7* deserves further investigation.

## Conclusions

In conclusion, this study characterized the transcription and expression of Notch pathway components (receptors, ligands and effectors) at the individual embryo level, during mouse blastocyst development and hatching. The transcription levels of Notch genes followed a dynamic pattern along development. Transcription levels of *Notch1*, *Notch2*, *Jagged2* and *Hes1* correlated with each other and with those of pluripotency and differentiation genes. Gene transcription was associated to protein expression, except for Jagged2, where high transcription levels in all embryos were not translated into protein, possibly reflecting mRNA storage for use at a later stage of development and/or interaction with the endometrium. Presence of Notch signaling activity was confirmed through nuclear NICD and Hes1 detection, and downregulation of *Hes1* transcription following canonical signaling blockade with DAPT. Data lead to the suggestion that Notch canonical signaling may be operating through Notch1, Notch3, Jagged1 and Hes1. In vitro embryo culture supplementation with Jagged1 had no effect on embryo developmental kinetics. In contrast, supplementation with Jagged2 abolished *Jagged1* transcription, downregulated *Cdx2* transcription and inhibited blastocyst hatching. This unveiled a possible regulatory effect between Jagged1, Cdx2 and blastocyst hatching. Notch signaling blockade by DAPT downregulated transcription of *Sox2*, and retarded embryo hatching. This indicates that a deregulation in Notch signaling, either by its over or under-activation, affects blastocyst development and hatching.

## Methods

### Animals

Animal manipulation and experimental procedures were conducted according to the national and European Union legislation regarding the use of animals for experimental purposes, and under the license of the national regulatory agency (DGAV – Direção Geral de Alimentação e Veterinária) and Institutional Animal Care and Use Committee (CEBEA – Comissão de Ética e Bem-Estar Animal; Ref. 001/2018). Male and female Crl: CD1 (ICR) (CD1) mice were purchased from Charles River Laboratoire France and maintained at the Faculty of Veterinary Medicine of the University of Lisbon animal house facilities. Mice were maintained in a 12 h light/dark cycle, in corn cob bedded cages and with ad libitum access to standard laboratory diet and water. Mouse health was monitored daily.

### Embryo collection and in vitro culture

Embryos were obtained from 2 to 3 months-old CD1 female mice, following superovulation and mating with CD1 males. Briefly, females were injected intraperitoneally with 10 IU equine chorionic gonadotropin (Intergonan; MSD Animal Health, Portugal) and 46 h later with 10 IU human chorionic gonadotropin (hCG; Chorulon; MSD Animal Health). Females were then housed overnight with a male and the presence of a vaginal plug was checked the following morning (0.5 dpc). At 2.5 dpc, females were euthanized by cervical dislocation under general anesthesia (intraperitoneal injection with 150 mg kg^− 1^ ketamine + 10 mg kg^− 1^ xylazine) and embryos were collected by oviduct flushing with M2 medium (Sigma-Aldrich, St Louis, MO, USA). Morphologically normal 8 to 16-cell embryos were selected, washed in M2 medium and in vitro cultured in groups of 20 in 500 μl of KSOM (Millipore, Specialty Media, Germany) overlaid with 400 μl of mineral oil (EmbryoMax®, Millipore), in 4-well dishes (Nunclon, Nunc, Roskilde, Denmark), at 37 °C in a 90% N_2_ + 5% O_2_ + 5% CO_2_ humidified atmosphere. Following a 24, 36 and 48 h culture (corresponding to respectively 3.5 dpc, 4.0 dpc and 4.5 dpc), embryos were classified into the CM, BL, EBL, eHBL (early HBL) and HBL developmental stages, according to Nagy et al. (2003) [44] (Fig. 6).

### Gene transcription analysis - qRT-PCR

Quantification of transcripts of Notch components – receptors (*Notch1*, *Notch2*, *Notch3* and *Notch4*), ligands (*Delta-like1* - *Dll1*, *Delta-like4* - *Dll4*, *Jagged1* and *Jagged2*), and effectors (*Hes1* and *Hes2*) – and of transcripts of pluripotency and differentiation gene markers *Sox2*, *Klf4*, *Oct4*, *Cdx2*, *Cdca7* and *Lgr5* was analyzed in individual 3.5 dpc CM (*n* = 9), BL (*n* = 9) and EBL (*n* = 7) and 4.5 dpc HBL (*n* = 5). Overall, transcription was individually evaluated in 30 embryos.

RNA extraction of single embryos was performed using the Arcturus® PicoPure™ RNA Isolation Kit (Applied Biosystems, ThermoFisher Scientific, USA) and DNA digestion with RNase-free DNase Set (Qiagen, Hilden, Germany). Concentration and purity of RNA were assessed spectrophotometrically at 260 and 280 nm (NanoDrop®2000c, ThermoFisher Scientific). Complimentary DNA (cDNA) synthesis was performed using Maxima First Strand cDNA Synthesis Kit for RT-qPCR (ThermoFisher Scientific) using 20 ng of total RNA in each reaction. Pre-amplification of cDNA was achieved with SSoAdvanced™ PreAmp Supermix (BioRad, CA, USA) using 10 μl of undiluted cDNA and a primer pool of genes *Notch1–4*, *Dll1* and *Dll4*, *Jagged1*–*2*, *Hes1*–*2*, *Sox2*, *Klf4*, *Oct4*, *Cdx2*, *Lgr5*, and reference genes *Rps29* and *Hprt1* (Table 3). With the exception of *Sox2*, which is coded by a single exon, primers were designed to bracket two exons to avoid genomic DNA amplification. In the case of *Sox2*, the cDNA specific amplification was confirmed with a minus-reverse transcriptase control.

**Table 3:** Primer sequences for target genes

Target gene	Sequence (5′ – 3′)	Product length (bp)	Accession no.
*Notch1*	Fwd: ACAGTAACCCCTGCATCCAC Rev.: GGTTGGACTCACACTCGTTG	120	NM_008714.3
*Notch2*	Fwd: GACTGCACAGAAGACGTGGA Rev.: GCGTAGCCCTTCAGACACTC	116	NM_010928.2
*Notch3*	Fwd: GTGTCAATGGTGGTGTCTGC Rev.: GCACACTCATCCACATCCAG	103	NM_008716.2
*Notch4*	Fwd: GAGGGACACTCCACCTTTCA Rev.: CTGGTGCCTGACACAGTCAT	93	NM_010929.2
*Delta-like1*	Fwd: GTTGTCTCCATGGCACCTG Rev.: TGCACGGCTTATGGTGAGTA	111	NM_007865.3
*Delta-like4*	Fwd: GGAACCTTCTCACTCAACATCC Rev.: CTCGTCTGTTCGCCAAATCT	141	NM_019454.3
*Jagged1*	Fwd: CCAGCCAGTGAAGACCAAGT Rev.: CAATTCGCTGCAAATGTGTT	127	NM_013822.5
*Jagged2*	Fwd: AGTGCCATCTGGCTTTGAAT Rev.: CGCTGCACATGGGTTAGAG	97	NM_010588.2
*Hes1*	Fwd: GCGAAGGGCAAGAATAAATG Rev.: TGTCTGCCTTCTCTAGCTTGG	104	NM_008235.2
*Hes2*	Fwd: CGGATCAACGAGAGCCTAAG Rev.: GTCTGCCTTCTCCAACTTCG	93	NM_001301805.1; NM_008236.4
*Sox2*	Fwd: GGTTCTTGCTGGGTTTTGATTCT Rev.: CCTTCCTTGTTTGTAACGGTCCT	59	NM_011443.4
*Klf4*	Fwd: GCAGTCACAAGTCCCCTCTC Rev.: GACCTTCTTCCCCTCTTTGG	93	NM_010637.3
*Oct4*	Fwd: TGGAGGAAGCCGACAACAAT Rev.: GCTGATTGGCGATGTGAGTG	177	NM_001252452.1; NM_013633.3
*Cdx2*	Fwd: CTGGCTCCGCAGAACTTTGT Rev.: GGTGCGTAGCCATTCCAGTC	170	NM_007673.3
*Cdca7*	Fwd: ACA TGC TGG TGA GAC AGA GGA A Rev.: TAT ATG CGG AAG GGT CAT GGA	98	NM_025866.3
*Lgr5*	Fwd: CCC ATC CAA TTT GTT GGA GTA Rev.: GTG GCA GTT CCT GTC AAG TG	113	NM_010195.2
*Rps29*	Fwd: CACGGTCTGATCCGCAAATAC Rev.: ACTAGCATGATCGGTTCCACTTG	144	NM_009093.2
*Hprt1*	Fwd: GTCGTGATTAGCGATGATGAACC Rev.: GCAAGTCTTTCAGTCCTGTCCATAA	128	NM_013556.2

Pre-amplified cDNA was diluted 1:10 in Tris-EDTA buffer and kept at − 20 °C until qRT-PCR analysis. This was performed in duplicate wells in StepOne Plus™ (Applied Biosystems, ThermoFisher Scientific) in 96-well optical reaction plates (Applied Biosystems), using the universal temperature cycles: 10 min of pre-incubation at 95 °C, followed by 40 two-temperature cycles (15 s at 95 °C and 1 min at 60 °C). Melting curves were acquired to ensure that a single product was amplified in the reaction. Each reaction used 10 μl of Perfecta® Sybr® Green Fast Mix, ROX™ (Quanta bio, MA, USA), 2 μl of diluted pre-amplified cDNA (corresponding to 0.2 ng of cDNA) and 80 nM of each primer in a total reaction volume of 20 μl. A NTC (no-template control) was included in all reaction plates and only plates with undetermined Ct in NTC wells were analyzed. Also, only wells with a single specific melting curve peak were analyzed. For each gene, one PCR product was run through a 2.5% agarose gel to confirm expected product size and the identity of this PCR product was confirmed by DNA sequencing. All reactions with the same Tm as the confirmed PCR product were considered specific. Positive controls were added to each reaction plate to exclude primer design artifacts: mouse uterus in oestrus for *Notch1*, *Dll4* and *Hes1* transcription, mouse uterus in metoestrus for *Notch2*, *Notch3* and *Hes2* transcription, mouse uterus in dioestrus for *Notch4*, *Jagged1* and *Jagged2* transcription [33], and mouse small intestine for *Dll1* and *Lgr5* [45]. Embryos themselves were used as positive controls for *Sox2*, *Klf4*, *Oct4*, *Cdx2* and *Cdca7* transcription [18, 19].

The first step in transcription data analysis was the calculation of prevalence among embryos, i.e. the proportion of embryos with detected transcription at each developmental stage. Genes with a Ct value > 35 were considered without amplification. This was further confirmed by visualization of qRT-PCR products in agarose gels (Table 1 and Fig. 1). The next step in transcription analysis was to quantify transcription levels of most prevalent genes. This was performed by two approaches. In Fig. 2 panel a, Ct values were normalized to housekeeping gene 1 (*Rps29*) and the ∆Ct values obtained further calibrated with housekeeping gene 2 (*Hprt1*), generating ∆∆Ct values. These values were log transformed and results presented as the Log_2_ of power of ∆∆Ct values. The Log_2_ of power of ∆Ct values of transcription levels of housekeeping genes *Rps29* and *Hprt1* at each developmental stage are shown in Fig. 2 panel b. The second approach is shown in Fig. 2 panel c. Here, Ct values of each target gene were normalized with the mean Ct values of housekeeping genes *Rps29* and *Hprt1*, and the obtained ∆Ct values were then calibrated to ∆Ct values of compact morulae (shown as 0.0), originating the ∆∆Ct values for log transformation [46]. Results are also presented as the Log_2_ of power of ∆∆Ct values.

### Gene expression analysis - immunocytochemistry

Embryos (3.5 dpc BL) were fixated in a 4% paraformaldehyde solution for 30 min, at 4 °C, permeabilized in phosphate-buffered saline (PBS) + 0.5% Triton X-100 for 1 min and washed in PBS. Blocking was performed in a PBS + 0.1% Tween20 solution containing 2.5% bovine serum albumin (Sigma-Aldrich) for 1 h at room temperature, followed by a 4 °C overnight incubation with the primary antibody diluted in blocking solution. Primary antibodies, all polyclonal and already validated for use in mouse cells [36, 47], were diluted as presented in Table 4. Negative IgG controls were performed using rabbit polyclonal IgG (ab27478, Abcam) and goat polyclonal IgG (ab37373, Abcam) at the appropriate dilutions. Embryos were then washed in PBS (4 × 5 min) and incubated with AlexaFluor® 594 chicken anti-rabbit (A11012, Life Technologies, USA) or chicken anti-goat (A21468, Life Technologies) secondary antibody diluted 1:300 in blocking solution, according to primary antibody host species, for 30 min, at room temperature. Embryos were then washed 2 × 10 min in PBS followed by Hoechst33268 (Sigma-Aldrich) nuclear labeling and finally mounted in ProLong™ Gold Antifade Mountant (Life Technologies). For each primary antibody, 6 blastocysts were analyzed, and a Z-stack was captured using a Zeiss LSM 710 confocal microscope (Carl Zeiss Microscopy, Oberkochen, Germany) with an optical magnification of 400× and treated with Fiji software (National Institutes of Health, USA).

**Table 4:** Immunocytochemistry primary antibodies, dilutions and manufacturer and catalogue reference, as used by Murta et al. [36, 47]

Antibody	Source	Dilution	Supplier (catalogue number)
anti-Notch1	Rabbit polyclonal	1:100	Abcam (ab8925)
anti-Notch2	Rabbit polyclonal	1:100	Abcam (ab8926)
anti-Notch3	Rabbit polyclonal	1:200	Abcam (ab23426)
anti-Notch4	Rabbit polyclonal	1:50	Santacruz Biotechnology (sc5594)
anti-Dll1	Rabbit polyclonal	1:100	Abcam (ab76655)
anti-Dll4	Rabbit polyclonal	1:200	Abcam (ab7280)
anti-Jagged1	Rabbit polyclonal	1:50	Santacruz Biotechnology (sc8303)
anti-Jagged2	Goat polyclonal	1:50	Santacruz Biotechnology (sc8158)
anti-Hes1	Rabbit polyclonal	1:100	Abcam (ab71559)
anti-Hes2	Rabbit polyclonal	1:100	Abcam (ab134685)

### Embryo culture supplementation with notch ligands and a notch signaling inhibitor

**Fig. 7:**
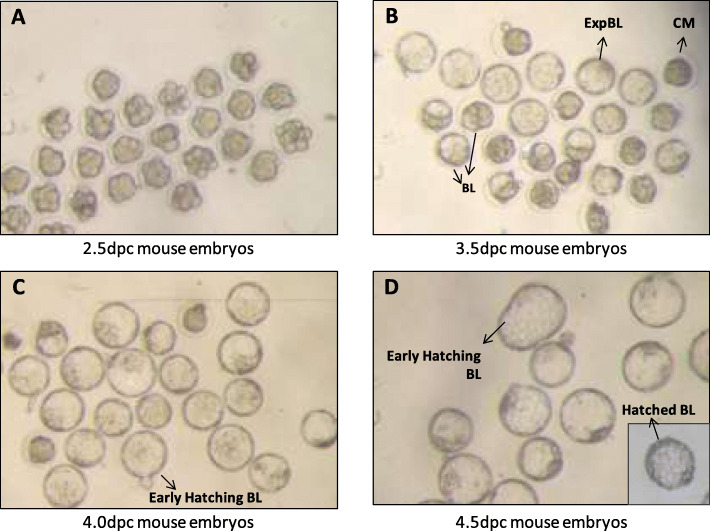
Representative photographs illustrating the morphological staging of embryonic development. Mouse embryos were in vivo collected at 2.5 dpc (**a**), in vitro cultured, and morphologically evaluated according to Nagy et al. 2003 [44] at 3.5 dpc (cultured for 24 h; **b**), 4.0 dpc (cultured for 36 h; **c**) and 4.5 dpc (cultured for 48 h; **d**). CM = compact morula, BL = blastocyst, EBL = expanded blastocysts, eHBL = early hatching blastocyst, HBL = hatched blastocyst

Mouse 8–16 cell embryos were collected and in vitro cultured as previously described, being randomly allocated in groups of 20 to each of the following treatment groups: i) Control, without treatment ii) Jagged1, medium supplemented with 1 μg ml^− 1^ Jagged1 (1277-JG, R&D Systems, Bio-Techne, USA); iii) Jagged2, medium supplemented with 1 μg ml^− 1^ Jagged2 (4748-JG, R&D Systems); and iv) DAPT, medium supplemented with 100 μM DAPT (N-[N-(3,5-Difluorophenacetyl)-L-alanyl]-S-phenylglycine t-butyl ester; Sigma-Aldrich). The experiment considered 10 in vitro culture sessions (550 embryos) until 3.5 dpc (24 h), from which 9 sessions (511 embryos) were further cultured until 4.0 dpc (36 h), and from the latter, 6 sessions (301 embryos) were further cultured until 4.5 dpc (48 h). Embryos were evaluated for viability, expressed as non-degenerated morphologically normal embryos progressing in culture, and their developmental stage recorded at those time-points (Fig. 7) by a technician blinded to group assignment, according to criteria established by Nagy et al. (2003) [44]. Five to six individual 4.0 dpc EBL from each group were processed for quantification of transcripts of Notch genes (*Notch1–2*, *Jagged1*–*2*, *Hes1*) and pluripotency and differentiation marker genes (*Sox2*, *Klf4*, *Oct4*, *Cdx2*, *Cdca7*), as described above.

### Statistical analysis

Statistical analysis was performed using the statistical software SPSS Statistics (version 22, IBM® SPSS® Statistics, 2013, IBM, NY, USA). Real-time PCR data (ΔCt values) did not follow normal distribution (Fig. S1) and were transformed to log 2 of power of ∆∆Ct for normalization, which allowed the use of parametric tests. Regarding *Notch1*, *Notch2*, *Jagged1*, *Jagged2*, *Hes1*, *Sox2*, *Oct4*, *Klf4* and *Cdx2* transcription, ANOVA was performed to compare the relative transcription between developmental stages, followed by LSD *post-hoc* analysis. Two-sided Pearson correlation coefficient was calculated to investigate the relationship between the transcription of Notch components, and between the latter and the transcription of pluripotency/differentiation markers. Chi-square test was used to evaluate the effect of Jagged1, Jagged2 and DAPT medium supplementation on in vitro cultured embryo viability and developmental rates. Results were considered significant if *p* < 0.05.

## Supplementary information

**Additional file 1: Figure S1.** Boxplot of ∆Ct values of transcription levels of Notch and pluripotency and differentiation genes in 3.5 dpc compact morulae (*n* = 9), blastocysts (*n* = 9) and expanded blastocysts (*n* = 7), and in 4.5 dpc hatched blastocysts (*n* = 5). Ct values of target genes were normalized to the average of Ct of housekeeping genes *Rps29* and *Hprt1*.

## Data Availability

The datasets used and/or analyzed during the current study are available from the corresponding author on reasonable request.

## Abbreviations

ANOVA: Analysis of variance
BL: Blastocyst
cDNA: Complimentary DNA
CM: Compact morula
Ct: Threshold cycle
dpc: Days post-coitum
eHBL: Early hatching blastocyst
EBL: Expanded blastocyst
HBL: Hatched blastocyst
ICM: Inner cell mass
mRNA: Messenger RNA
NICD: Notch intra cellular domain
qRT-PCR: Quantitative real-time PCR
s.e.m.: Standard error of the mean
TE: Trophectoderm
