# Supplementary material for: Notch signaling in mouse blastocyst development and hatching
Source: BMC Dev Biol. 2020 Jun 2;20:9. doi: 10.1186/s12861-020-00216-2 (PMC7265256; doi:10.1186/s12861-020-00216-2)
Supplement: Supplementary file 1 — Additional file 1: Figure S1. Boxplot of ∆Ct values of transcription levels of Notch and pluripotency and differentiation genes in 3.5 dpc compact morulae (n = 9), blastocysts (n = 9) and expanded blastocysts (n = 7), and in 4.5 dpc hatched blastocysts (n = 5). Ct values of target genes were normalized to the average of Ct of housekeeping genes Rps29 and Hprt1. [file 12861_2020_216_MOESM1_ESM.docx]

**S Figure1**
